# CYP51A1 polymorphism and voriconazole-associated hepatotoxicity in children undergoing hematopoietic cell transplant 

**DOI:** 10.5414/CP203920

**Published:** 2021-02-09

**Authors:** Takuto Takahashi, Angela R. Smith, Pamala A. Jacobson, Abeer F. Alharbi, James Fisher, Nathan T. Rubin, Mark N. Kirstein

**Affiliations:** 1 Division of Hematology and Oncology,; 2 Division of Blood and Marrow Transplant, Department of Pediatrics,; 3 Department of Experimental and Clinical Pharmacology, and; 4 Department of Masonic Cancer Center Biostatistics Core, University of Minnesota, Minneapolis, MN, USA

**Keywords:** CYP51A, pharmacogenomics, voriconazole, hepatotoxicity

## Abstract

Fungal *CYP51A* (14α-sterol demethylase) is the target of an azole antifungal, voriconazole (VCZ), which also partially inhibits human *CYP51A1*. Hepatotoxicity is a common adverse effect of azoles, which is reported to be caused by altered gene expressions secondary to cholesterol synthesis inhibition by azoles. This is a post-hoc analysis of a previously conducted phase 1 dose-finding study of prophylactic VCZ in 56 pediatric hematopoietic cell transplant recipients. We explored an association between variants in human *CYP51A1* (rs2282976 and rs6465348) and VCZ-induced hepatotoxicity. Genotype A/G or G/G in rs6465348 showed lower odds of hepatotoxicity after adjusting for VCZ area-under-the-curve (OR: 0.10, 95% CI: 0.01 – 0.79, vs. A/A).

**What is known about this subject**

- CYP51A is the fungal protein targeted by voriconazole, and its homolog mediates cholesterol synthesis in humans. Azole antifungals affect the regulatory genes involved in cholesterol synthesis, which results in azole-induced hepatotoxicity in patients.

**What this study adds**

- The single nucleotide variant of rs6465348 located in CYP51A1 gene may render protection against voriconazole-induced hepatotoxicity among pediatric hematopoietic-cell transplantation recipients.

## Introduction 

Voriconazole (VCZ) is an azole antifungal agent with a narrow therapeutic index used for prophylaxis in highly immunocompromised patients. Hepatotoxicity is among the most common adverse effects of azole antifungals (> 5%), especially when trough concentrations exceed 5 mg/L [1]. To date, genetic risk factors of VCZ hepatotoxicity have not been reported. 

VCZ inhibits fungal CYP51A (14α-sterol demethylase), which mediates ergosterol synthesis, whereas its human homolog is a key enzyme in cholesterol biosynthesis pathway. An in vitro model utilizing purified human and fungal CYP51A protein showed that azoles bind fungal CYP51A protein with a reported dissociation constant of 10 nM and the human CYP51A1 homolog, 2,290 nM [2]. A recent study suggested that altered gene expression driven by impaired cholesterol synthesis led to hepatotoxicity after azole exposure [3]. 

We hypothesized that variable susceptibility to azole-induced inhibition of cholesterol synthesis pathways may affect the risk of azole-induced hepatotoxicity. In this study, we explored our hypothesis that genetic polymorphisms of CYP51A1 alter the risk of VCZ-induced hepatotoxicity in pediatric hematopoietic stem cell (HCT) recipients receiving prophylactic VCZ therapy. 

## Materials and methods 

### Study setting 

We conducted a post-hoc analysis of pediatric HCT patients aged ≤ 21 years enrolled in a single-institution, phase I dose-finding study of intravenous VCZ (NCT02227797) in pediatric HCT recipients conducted at the University of Minnesota from 2015 to 2017. Children with pre-existing kidney or hepatic dysfunction were not eligible to participate in the study. The details of the study’s methodology were published previously [4]. A total of 58 participants with evaluable VCZ pharmacokinetic and the genotype data, of the 65 study participants, were included in this analysis (i.e., 6 had no pharmacokinetic data, and 1 no genotype data). The study was approved by the University of Minnesota Institutional Review Board and the Masonic Cancer Center Protocol Review Committee, and all participants and/or parents or guardians provided informed consent. 

### Outcomes 

The primary outcome was VCZ-induced hepatotoxicity, which was defined as a liver dysfunction of any grade attributed to VCZ within the first week of its use according to the Common Terminology Criteria for Adverse Events version 4, namely, total bilirubin level > 1.5-fold of the upper limit of normal (ULN), or AST or ALT > 3-fold ULN. Liver dysfunction was monitored at least twice a week. 

### Pharmacokinetic analysis 

Varying doses of prophylactic intravenous VCZ (3 mg/kg/hour, every 12 hours) were initiated on the day after stem cell infusion (i.e., HCT). Intensive observational pharmacokinetic (PK) studies of VCZ were performed on days 5 – 7 including six timepoints (i.e., 5 and 30 minutes; 1, 3, 6, and 9 hours) after infusion completion and immediately before the next infusion start. All concentrations of VCZ were above the limit of quantitation (0.025 mg/L) as measured by an LC-MS/MS assay [5]. A pharmacokinetic assessment of steady-state profiles was performed with non-compartmental analysis with a trapezoidal method by linear-up-log-down method as implemented in R (version 3.6.0) PKNCA package (version 0.8.5). If the last timepoint plasma specimens (i.e., immediately before the next dose) were not collected at precisely 12 hours after the start of the infusion, we estimated the last timepoint VCZ concentrations by extrapolating/interpolating concentrations based on linear best-fit regression estimates for elimination rates from at least 3 concentration timepoints closest to 12 hours. We excluded patients from analyses when their inter-/extrapolated VCZ area-under-the-curve (AUC) was > 20% of the AUC without inter-/extrapolation. Consequently, we excluded 2 patients, which led to a final study population of 56 patients. 

### Genotyping of candidate SNPs 

Pre-transplant recipient germline genomic DNA extracted from the blood was examined by a custom Amplicon SNP panel at the University of Minnesota Genomics Center. CYP2C19 phenotype was determined according to the Clinical Pharmacogenomics Implementation Consortium guidelines [6]. We selected two SNPs in the *CYP51A1* gene with a minor allele frequency of > 10%: rs6465348 (chr7:92113288, A>G, 3’-UTR variant) and rs2282976 (chr7:92132811, A>G, intron variant). Samples without a genotype call were excluded from the analyses (4 of 56 for rs6465348 and 6 of 56 for rs2282976). The minor allele frequencies were 39.4 and 4.0% for rs6465348 and rs2282976, respectively, and were both in Hardy-Weinberg equilibrium (p = 0.77 and 1.00, respectively). We reveal no relevant variants with either SNP in haplotype analysis. 

### Statistical analysis 

Univariate analysis was performed by 2-sample t-test for numerical and χ^2^-test for categorical variables. Multivariate logistic regression analysis was performed to assess the association of *CYP51A1* SNPs and liver toxicity of any grade, while adjusting for VCZ AUC (treated as a continuous variable). Firth method was used because of the small event proportion in our cohort. Without any information on the clinical consequence of the variants, we tested the effect of the variants both in dominant and recessive models. rs2282976 is tested in a recessive model because there were no patients with homozygous minor alleles. All statistical analyses are conducted by using R software (version 3.5.3). 

## Results 

Of the 56 children in the final analysis, the mean age was 9.4 years (SD 6.5), and 64% were male (Table 1). Hepatotoxicity of any grade was observed in 7 patients (13%); 2 with grade 3 increased AST and/or ALT, and 5 with isolated hyperbilirubinemia (median grade 3, range 1 – 3). The most common HCT indication was acute leukemia (48%). No significant association was observed on hepatotoxicity by age, gender, CYP2C19 phenotype, disease, or VCZ AUC by univariate analysis (p = 0.34, 0.67, 0.95, 0.21, and 0.24, respectively) (Table 1). In the multivariate logistic regression model for rs6465348 genotypes adjusting for VCZ AUC, the variants in the dominant model showed significantly lower odds of hepatotoxicity in comparison to the homozygous wild-allele (A/G or G/G vs. A/A; odds ratio: 0.10, 95% CI: 0.01 – 0.79, p = 0.03). In this model, VCZ AUC was approaching significance with toxicity (odds ratio: 1.02, 95% CI: 1.00 – 1.05, p = 0.06) (Table 2). No significant association was observed between rs6465348 recessive model or rs2282976 and VCZ AUC (p = 0.25 and 0.84, respectively) in the logistic regression model. 

## Discussion 

The present study suggested a novel protective effect of rs6465348 variant in *CYP51A1* from VCZ-induced hepatotoxicity among pediatric HCT recipients. rs6465348 is a variant located in the 3’-untranslated region of *CYP51A1*, which encodes 14α-sterol demethylase in human cholesterol biosynthesis pathways. No prior published studies have reported the association of rs6465348 and VCZ-induced toxicity. This SNP has been associated with the incidence of low birth weight in newborns and lower cholesterol levels in mothers; this report indicates functional alterations of CYP51A on the cholesterol pathway despite its locus being in the non-coding region [7]. 

We speculate that potential resistance against VCZ-induced cholesterol synthesis inhibition can explain the observed protective effect of the rs6465348 variant in 3’-untranslated region of CYP51A1. Although we found no data specific to CYP51A1 in the literature, 3’-untranslated regions generally serve as a binding site for microRNA, which downregulates the target gene expression. Thus, alteration in this region can inhibit the binding of microRNA and subsequently lead to increased gene expression. It may be possible that increased production of CYP51A1 protein in the rs6465348 variant can make individuals less prone to impairment effect on cholesterol synthesis secondary to CYP51A1 inhibition by VCZ. This protection against azole-induced cholesterol pathway suppression can consequently prevent azole-induced hepatotoxicity because of their interconnection by gene regulations. A gene expression analysis, using in-vivo and in-vitro mouse models for azole-induced hepatotoxicity, suggested that cholesterol synthesis inhibition upon exposure to azoles lead to compensatory overexpression of upstream regulatory genes for cholesterol pathway; these genes activate pathways involved in oxidative stress and cytokine production, which lead to hepatotoxicity [3]. 

A main limitation of this study is the small sample size. However, the use of VCZ AUC from an intensive PK study allowed an adjustment of variable VCZ exposure, which would otherwise be a major confounder in studies with a small sample size. This study was a hypothesis-driven exploratory study and did not assess the involvement of other potential causes of drug-induced hepatotoxicity (e.g., toxic metabolites, mitochondrial dysfunction, or apoptosis) [8]. 

We report a potential novel link between a *CYP51A1* variant (rs6465348) and a lower incidence of hepatotoxicity with VCZ use. Future research should focus on validation of our findings in large patient cohorts with various demographic and disease characteristics, functional analysis of rs6465348, and exploration of its clinical utility. 

## Acknowledgment 

We would like to acknowledge the assistance of the Clinical Pharmacology Shared Resource of the Masonic Cancer Center, designated by the National Cancer Institute, supported in part by P30 CA77598. 

## Funding 

This work was supported by the Hematology Oncology Pharmacist Association (HOPA) Foundation (MNK), and the Department of Pediatrics, Division of Blood and Marrow Transplant, University of Minnesota (ARS). 

## Conflict of interest 

Nothing to declare. 

**Table 1.** Patient characteristics and univariate analysis.

	Total	Hepatotoxicity		p-value
		Absent	Present	
	(n = 56)	(n = 49)	(n = 7)	
Age (years), mean (SD)	9.4 (6.5)	9.1 (6.6)	11.7 (5.6)	0.33
Male sex, n (%)	36 (64%)	31 (63%)	5 (71%)	1.00
CYP2C19 phenotype, n (%)				0.95
Poor	1 (2%)	1 (2%)	0	
Intermediate	13 (23%)	11 (33%)	2 (29%)	
Normal	29 (52%)	26 (53%)	3 (43%)	
Rapid	12 (21%)	10 (30%)	2 (29%)	
Ultra-rapid	1 (2%)	1 (2%)	0	
rs6465348				0.14
A/A	19 (34%)	14 (39%)	5 (71%)	
A/G	25 (45%)	23 (47%)	2 (29%)	
G/G	8 (14%)	8 (16%)	0	
No call^a^	4 (7%)	4 (8%)	0	
rs2282976				0.17
A/A	46 (82%)	42 (86%)	4 (57%)	
A/G	4 (7%)	3 (6%)	1 (14%)	
No call^a^	6 (11%)	4 (8%)	2 (29%)	
Drug-drug interactions, n (%)				
Pantoprazole	52 (93%)	45 (92%)	7 (100%)	1.00
Corticosteroids	5 (9%)	5 (10%)	0	0.86
Drugs listed in FDA label^b^	0	0	0	.
Indications, n (%)				0.99
Acute leukemia	25 (45%)	22 (45%)	3 (43%)	
Aplastic anemia	8 (14%)	7 (14%)	1 (14%)	
Myelodysplastic syndrome	6 (11%)	5 (10%)	1 (14%)	
Others	17 (30%)	15 (31%)	2 (29%)	
VCZ dose (mg/kg), mean (SD)	8.2 (2.0)	8.3 (2.0)	7.8 (1.9)	0.50
VCZ trough (mg/L), mean (SD)	1.9 (1.9)	1.9 (1.9)	1.7 (1.8)	0.79
VCZ AUC (mg×h/L), mean (SD)	42.1 (35.9)	40.4 (33.2)	54.5 (52.4)	0.33
VCZ AUC, dose normalized (mg×h/L)/(mg/kg), mean (SD)	5.2 (4.0)	5.1 (3.8)	6.5 (5.2)	0.37

SD = standard deviation; FDA = the US Food and Drug Administration; VCZ = voriconazole; AUC = area-under-the-curve. ^a^No reliable genotype call was made by the laboratory; ^b^includes the drugs listed under “Drug Interactions: Effects of other drugs on voriconazole” in the FDA package insert.

**Table 2.** Logistic regression analyses on hepatotoxicity.

		Hepatotoxicity	
Predictors	Odds ratio	95% CI	p-value
Model 1 (rs6465348: A>G, dominant)			
rs6465348, A/G or G/G (vs. A/A)	0.08	(0.004 – 0.58)	0.03
VCZ AUC (mg×h/L)	1.02	(0.997 – 1.05)	0.08
Model 2 (rs6465348: A>G, recessive)			
rs6465348, G/G (vs. A/A or A/G)	0.00	NA*	0.99
VCZ AUC (mg×h/L)	1.01	(0.99 – 1.03)	0.35
Model 3 (rs2282976: A>G, recessive)			
rs2282976, A/G (vs. A/A)	3.83	(0.16 – 43.34)	0.30
VCZ AUC (mg×h/L)	0.99	(0.94 – 1.02)	0.56

VCZ = voriconazole; AUC = area under the curve. *Not estimated because no event observed in G/G group.
